# Biofabrication of *Terminalia ferdinandiana*-Conjugated Gold Nanoparticles and Their Anticancer Properties

**DOI:** 10.3390/life15121829

**Published:** 2025-11-28

**Authors:** Weerakkodige Hansi Sachintha Alwis, Vinuthaa Murthy, Hao Wang, Roshanak Khandanlou, Richard Weir

**Affiliations:** 1 Faculty of Science and Technology, Charles Darwin University, Darwin, NT 0810, Australia; hansi.alwis@cdu.edu.au (W.H.S.A.); hao.wang@cdu.edu.au (H.W.); roshanak_bch@yahoo.com (R.K.); 2 Berrimah Veterinary Laboratory, Department of Agriculture and Fisheries, Darwin, NT 0801, Australia; richard.weir@nt.gov.au

**Keywords:** Kakadu plum, phytochemicals, green nanoparticles, antioxidant, cytotoxicity, Australian native plants, cancer cells, MCF-7 cells, MCF10A cells, HeLa cells

## Abstract

Harnessing nature’s chemistry, this study explores the enhanced biomedical potential of *Terminalia ferdinandiana* Exell (Kakadu Plum) by transforming its aqueous leaf and fruit extracts into bio-inspired gold nanoparticles (AuNPs). The synthesis process was optimized by varying the Au^3+^/extract ratio and pH, with nanoparticle formation verified through UV–visible spectrophotometry, TEM, and DLS analyzes. Kakadu Leaf extract–conjugated AuNPs (AuKLs), synthesized at pH 8 with a 1:25 Au^3+^/extract ratio, produced the smallest and most uniform particles (21.1 nm; PDI 0.17). In contrast, fruit extract alone failed to generate stable nanoparticles, highlighting the pivotal role of leaf phytochemicals as natural reducing and stabilizing agents. Biological evaluations revealed that both the crude leaf extract and AuKLs possessed strong antioxidant capacity, while the AuKLs further exhibited selective anticancer activity effectively inhibiting breast cancer (MCF-7) and human cervical carcinoma (HeLa) cell proliferation without harming normal mammalian breast (MCF10A) cells. A combined 2:1 leaf-to-fruit extract formulation yielded well-stabilized AuNPs (AuKPLs) with biomedical properties comparable to AuKLs, though the fruit extract alone contributed minimally to both nanoparticle formation and biological performance. Overall, this study demonstrates that the phytochemical richness of *T. ferdinandiana* leaves enables the green synthesis of small, stable, and bioactive gold nanoparticles. The resulting nanoconjugates, AuKLs and AuKPLs, hold considerable promise for future pharmacological and therapeutic applications, bridging traditional plant-based medicine with modern nanotechnology.

## 1. Introduction

Among the different types of nanoparticles, AuNPs possess tuneable sizes, multifunctionality, and biocompatibility, making them highly suitable for diverse applications such as drug delivery, bioimaging, and therapeutic interventions, as well as catalysts in chemical reactions [1,2,3,4,5,6,7,8]. For instance, Faid et al. [9] demonstrated the use of AuNPs as a drug carrier for the chemotherapeutic drug doxorubicin to cancer cells, and the AuNP-doxorubicin nanocomposite showed increased cytotoxicity on the MCF7 breast cancer cell line compared to free doxorubicin. This dual functionality of AuNPs enhances their effectiveness in targeting cancer cells [10,11] while minimizing the side effects associated with conventional chemotherapy, making them a promising avenue for future cancer treatments.

In our previous study [12], we reported the synthesis of well-defined silver nanoparticles (AgNPs) utilizing *T. ferdinandiana* extracts and their improved antimicrobial activities. In the current study, we introduce the benefits of synthesizing gold nanoparticles (AuNPs) using *T. ferdinandiana* extracts, emphasizing their potential anticancer properties.

The green synthesis method [12] was used to synthesize AuNPs as it employs environmentally friendly techniques using natural resources [13,14]. This green method does not use any organic solvents or chemicals of traditional methods, which often involve hazardous chemicals and generate toxic by-products, posing environmental and health risks [14,15]. Thus, the green synthesis of AuNPs using plant extracts involves the reduction of Au ions (Au^3+^) to AuNPs (Au^0^) using the natural reducing and capping agents present in plant extracts [13]. Plant extracts contain a wide array of bioactive compounds, such as polyphenols, flavonoids, tannins, and terpenoids, which exhibit significant reducing properties [16] that can effectively reduce metal ions during nanoparticle synthesis [17] by donating electrons, leading to the formation of nanoparticles [18]. For example, Lee et al. [19] successfully synthesized AuNPs using the flavonoid quercetin as a reductant in a green synthesis approach, highlighting its potential in nano-architectonic applications. Additionally, some phytochemicals found in plants play a crucial role as capping and stabilizing agents [16]. These biomolecules adhere to the surface of nanoparticles, preventing aggregation and improving stability. The presence of capping agents also impacts the size, shape, and surface properties of the synthesized nanoparticles, contributing to their overall stability and functionality [16,20]. Researchers have shown substantial interest in using green synthesized AuNPs for cancer therapy and drug delivery. Numerous studies have proven the anticancer efficacy of green-synthesized AuNPs utilizing various plant extracts [21,22,23,24] against a wide range of cancer cell types, such as breast cancer [21,25,26], melanoma [26], human liver cancer [25,26,27], lung carcinoma [27], bladder cancer [28], human prostate cancer [29], and human gastric carcinoma [30].

The work in this study aimed to produce environmentally friendly small, well-stabilized AuNPs by utilizing phytocomponents present in *T. ferdinandiana* leaves and fruits. As discussed in the previous paper [12], several studies have revealed the in vitro antibacterial [29,31], antioxidant [32], anti-inflammatory [33], and anticancer [29,34] properties of *T. ferdinandiana*. Where our previous research [12] indicated that the AgNPs conjugated with *T. ferdinandiana* extracts have significant antibacterial activity, in this study, we concentrate on the synthesis of AuNPs to evaluate their anticancer properties.

However, to the best of our knowledge, the *T. ferdinandiana*-mediated green synthesis of AuNPs has not yet been reported. The effects of different parameters, such as pH and Au^3+^ ion/aqueous extract ratio, on the synthesis of small, well-stabilized AuNPs, were investigated. The synthesized AuNPs were evaluated for their antioxidant properties and anticancer activity on human cervical carcinoma (HeLa) cells, breast cancer (MCF7) cells, and normal mammalian breast (MCF10A) cells.

## 2. Materials and Methods

### 2.1. Material

Fresh leaves and fruits of *T. ferdinandiana* were collected from Charles Darwin University (CDU), NT, (12°36′91” S, 130°86′68″ E), Australia. The voucher specimen is deposited at the Northern Territory Herbarium in Darwin, NT (D0293019 [Weerakkodige, H.A. s.n.]). Tetrachloroauric(III) acid trihydrate, (99.9%) (HAuCl_4_.3H_2_O), dimethyl sulfoxide (DMSO) (D8418), and AR-Grade methanol were purchased from Sigma-Aldrich, Melbourne, Victoria (VIC), Australia. 2,2-diphenyl-1-picrylhydrazyl (DPPH) and 3-(4,5-dimethylthiazol-2-yl)-2,5-diphenyl tetrazolium bromide (MTT) were purchased from Thermo Fisher Scientific, Scoresby, VIC, Australia. Cell lines, including MCF7 human breast adenocarcinoma cells, were purchased from Merck Life Science Pty Ltd., Macquarie Park, NSW, Australia. HeLa cells were obtained from the Berrimah Veterinary Laboratory, NT, Australia, and MCF10A cells were obtained from the NICM Health Research Institute, Western Sydney University, NSW, Australia. Modified Eagle’s Medium (MEM), the Roswell Park Memorial Institute (RPMI) 1640 Medium, and Phosphate-buffered saline (PBS) were purchased from Sigma-Aldrich, Burlington, MA, USA. The mammary epithelial cell basal medium (MEBM) was purchased from Lonza, Norwest, NSW, Australia.

### 2.2. Preparation of the Leaf and Fruit Extract

The fresh leaves and fruit of *T. ferdinandiana* were washed with high-purity water (HPW) until no foreign material remained, then stored in a freezer (−18 °C) until processed. The extraction of *T. ferdinandiana* fruit and leaf was performed according to the method described in previous work [12]. Both extracts were vacuum filtered through Whatman No. 1 filter paper (purchased from Thermo Fisher Scientific, Parkville, VIC, Australia), followed by 0.45 µm filtration (Sartorius Australia Pty. Ltd., Dandenong South, VIC, Australia). The filtrates were kept at 4 °C for future studies. The qualitative analysis of the major phytochemicals in *T. ferdinandiana* was performed using a UPLC-MS (ultra-performance liquid chromatography-mass spectrometry) system. The detailed analytical procedures and conditions for the UPLC-MS analysis are presented in the previous paper [12].

### 2.3. The Optimization Process of AuNPs

*T. ferdinandiana* fruit or leaf extracts were mixed with an aqueous solution of HAuCl_4_ to synthesize AuNPs. The reaction mixture was stirred for 30 min and then incubated overnight at room temperature.

Similarly to our synthesis of AgNPs [12], the optimal conditions for the biosynthesis of *T. ferdinandiana* AuNPs were investigated by examining the ratio of Au ions/extract (*w*/*w*) and the pH value of the reaction mixture (ranging from 4 to 10). Five different ratios of Au ions/extract (1:20, 1:25, 1:33, 1:50, and 1:100 in mg) were evaluated by combining the extracts with a 5.0 mM HAuCl_4_ solution. The mass ratios represent the mass of Au^3+^ in the 5 mM HAuCl_4_ solution in relation to the mass of the plant extract. The completion of the synthesis of nanoparticles was monitored by visualization of the colour change (from yellow to deep red), indicating the reduction process of Au^3+^ to Au^0^ nanoparticles. Additionally, AuNPs synthesized using a mixture of leaf and fruit extracts were prepared using a similar methodology.

### 2.4. Characterization of Gold Nanoparticles

The mean hydrodynamic diameter, PDI (polydispersity index), and UV-Vis (ultraviolet-visible) spectra were measured to determine the optimal conditions for synthesizing small and homogenized AuNPs. UV-Vis spectroscopy (Varian, Cary 100) Agilent Technologies, VIC Australia, was employed to confirm the formation of AuNPs, with spectra recorded at wavelengths ranging from 300 nm to 800 nm. In order to maintain the absorbance of AuNPs within the 0.1 to 1.0 range, each sample was diluted with HPW at a ratio of 1:100, before measurement. The mean hydrodynamic diameter, size distribution, and zeta potential of the AuNPs were measured using a Nanoparticle Analyzer SZ-100 (Horiba, Kyoto, Japan). The zeta potential was measured to assess the surface charge of the AuNPs, which is indicative of their stability in solution [35]. To prevent inter-particle interactions and multiple scattering, samples were diluted with HPW at a ratio of 1:100. The particle size and microscopic morphology of the AuNPs were examined using a JEOL 2100 TEM operating at 120 kV, conducted at the Queensland University of Technology (QUT), Brisbane, Queensland (Qld), Australia.

### 2.5. Antioxidant Activity Assay

The antioxidant potential of the *T. ferdinandiana* extracts and synthesized AuNPs was evaluated using a DPPH (2,2-diphenyl-1-picrylhydrazyl) radical scavenging assay, following a previous method [12]. Briefly, 1 mL of each methanolic sample of *T. ferdinandiana* extracts and AuNPs, prepared at concentrations ranging from 0.1 to 0.9 mg/mL (expressed in terms of extract content), was combined with 1 mL of a 1.0 mM DPPH methanolic solution.

Percentage inhibition of DPPH oxidation was calculated using Equation (1).

(1) $$DPPH\;scavenging\;effect\left(\%\right)=\left(\frac{A_{control}-A_{sample}}{A_{control}}\right)\times 100$$

where $A_{control}$ is the absorbance of the DPPH solution and $A_{sample}$ is the absorbance of the test sample.

### 2.6. Anticancer Assays

The three cell lines used for the study, MCF7, HeLa, and MCF10A, were grown in different culture media. The MCF7 cells were grown in Modified Eagle’s Medium (MEM) supplemented with 2.2 g/L L-glutamine, 10.0% fetal bovine serum (FBS), and 1% antibiotic/antimitotic (100 units of penicillin, 0.1 mg of streptomycin and 0.25 µg of amphotericin). The HeLa cells were cultured in Roswell Park Memorial Institute (RPMI) 1640 Medium with 2.2 g/L L-glutamine, 10.0% fetal bovine serum (FBS) and 1% antibiotic/antimitotic (100 units of penicillin, 0.1 mg of streptomycin, and 0.25 µg of amphotericin). The MCF10A cells were cultured in Mammary Epithelial cell Basal Medium (MEBM) supplemented with mammary epithelial cell growth medium SingleQuots kit; 500 mL of medium containing 2 mL of bovine pituitary extract, 0.5 mL of human epidermal growth factor, insulin (0.1%), 0.5 mL of hydrocortisone (0.1%), and 0.5 mL of gentamicin-amphotericin (GA-1000; 0.1%). All cells were incubated at 37 °C in the presence of 5% CO_2._

A MTT (3-(4,5-dimethylthiazol-2-yl)-2,5-diphenyltetrazolium bromide) assay was carried out to study the in vitro anticancer activity of *T. ferdinandiana* plant extracts and AuNPs on MCF7, HeLa, and MCF10A cells using the established method reported by previous researchers [36]. For MTT analysis, 150 μL of 1 × 10^5^ suspended cells were seeded into flat-bottom 96-well plates and incubated for 72 h at 37 °C. The cells were then treated with different concentrations (0.156–5.000 mg/mL, in terms of dry extract) of 150 μL of each extract and AuNPs and incubated for 24 h at 37 °C. Following a 24 hr incubation period, the medium containing the extract and AuNPs was removed. Further, the wells were washed with warm (37 °C) phosphate-buffered saline (PBS) to ensure the removal of traces of the coloured extract. Then, 20 μL of 5 mg/mL of MTT was added to each well and incubated for another four hours. The purple formazan crystals that formed were dissolved by adding 100 μL of 100% DMSO into each well. The absorbance was recorded with an ELISA microplate reader (xMark™, BIO-RAD, Gladesville, NSW, Australia) at 570 nm, and optical density (OD) was used to determine the cell viability. The percentage cell viability the of cells was calculated using Equation (2):

(2) $$Cell\;viability\;(\%)=\frac{A_{sample}}{A_{control}}\times 100$$

where $A_{control}$ is the absorbance of untreated cells and $A_{sample}$ is the absorbance of cells treated with samples. All assays were conducted in triplicate, and the experiment was repeated across three independent trials.

### 2.7. Statistical Analysis

The results of the biological studies were analyzed using GraphPad Software 10 (USA). Three independent experiments were conducted, each with internal triplicates, and the values are expressed as mean ± SD. Two-way analysis of variance (ANOVA) revealed significant differences at *p* < 0.05.

## 3. Results

### 3.1. Optimization and Characterization of AuNPs

The rapid formation of AuNPs using an extract of *T. ferdinandiana* by mixing the extract with an aqueous HAuCl_4_ solution at room temperature was evidenced by a distinct colour change in the solution from pale yellow to ruby red, indicating the reduction of Au^3+^ ions to Au^0^ atoms. The ruby-red colour observed in the aqueous solution is characteristic of AuNPs and is attributed to the excitation of surface plasmon resonance (SPR) vibrations [37].

**Figure 1:**
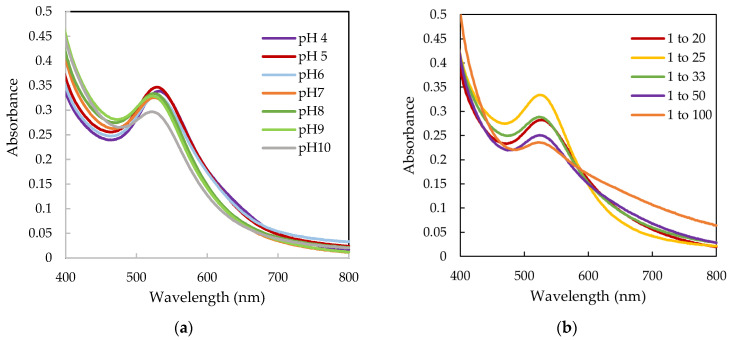
Ultraviolet-visible spectra of gold nanoparticles (Au) synthesized with leaf extract (AuKL) at (**a**) different pH values, and (**b**) different ratios of Au^3+^/leaf extract (*w*/*w*) at pH 8.

**Figure 2:**
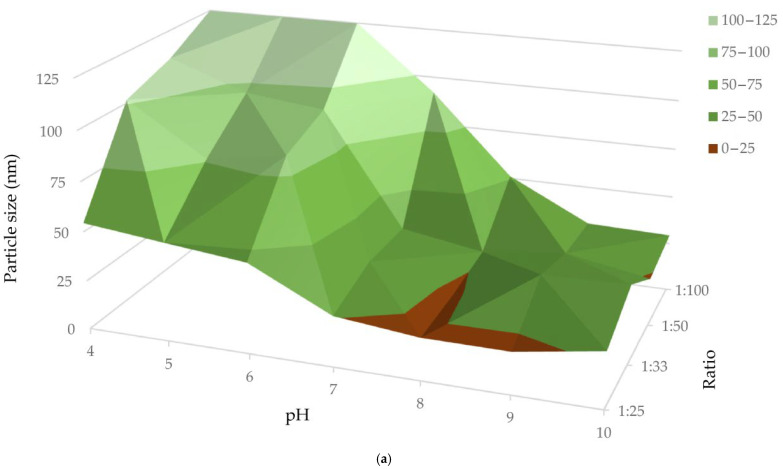

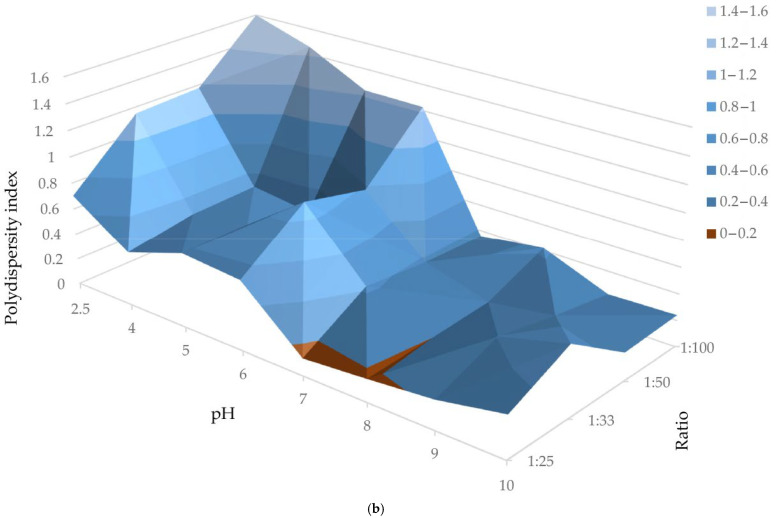
(**a**) Average particle size (nm) of gold nanoparticles (Au) synthesized with leaf extract (AuKLs) at different pH values and different Au^3+^/leaf extract ratios (*w*/*w*). (**b**) Polydispersity index of gold nanoparticles (Au) synthesized with leaf extract (AuKLs) at different pH values and different Au^3+^/leaf extract ratios (*w*/*w*).

The results obtained from UV-Vis and dynamic light scattering (DLS) for the synthesis formation of AuNPs with leaf extract (AuKLs) are shown in Figure 1 and Figure 2, respectively.

Figure 1a,b illustrate the effect of pH and the ion-to-extract ratio (*w*/*w*) on the formation of AuKLs. The absorption peaks observed in Figure 1a between 523 and 532 nm confirm the formation of AuNPs with the leaf extract. At pH 4, the primary absorption peak for AuKL synthesis was at 532 nm. As the pH increased from 4 to 10, a slight blue shift in the absorption spectra was noted, with the wavelength decreasing from 532 nm to 523 nm. The absorption peaks for pH levels 7 to 9 were nearly identical, appearing around 524 nm. However, at pH 10, the absorption spectra were broader, indicating a wider distribution of particle size. The UV-Vis spectra of AuKLs at different ratios, shown in Figure 1b, indicate an increase in the intensity of the absorption peaks as the amount of *T. ferdinandiana* leaf extract decreases, up to a Au ion-to-leaf extract ratio of 1:25. At this ratio, the AuKL nanoparticles exhibit a comparatively narrow SPR band centred at 524 nm, with the highest recorded intensity.

Figure 2a,b present three-dimensional plots of the DLS results, illustrating the particle size and PDI across various pH values and ion-to-extract ratios of synthesized AuKLs. These figures demonstrate that larger particle sizes and higher PDI values were obtained when synthesizing AuKLs at acidic pH compared to basic pH. The dark brown regions in both figures correspond to the smallest particle sizes, ranging from 0 to 25 nm, with a PDI between 0.0 and 0.2. The optimal condition for forming stable AuKL nanoparticles is at pH 8, which yielded a particle size of 2.1 nm and a PDI of 0.17.

An increase in particle size and PDI is observed with an increase in the amount of leaf extract added during the synthesis of AuKLs. Furthermore, the analysis of PDI and particle size, as indicated by the dark brown regions in Figure 2a,b, reveals that AuKLs synthesized at a 1:25 ion-to-leaf extract (*w*/*w*) ratio achieved the smallest particle size (2.1 nm) and lowest PDI (0.17) values, indicating uniform particle size distribution.

**Figure 3:**
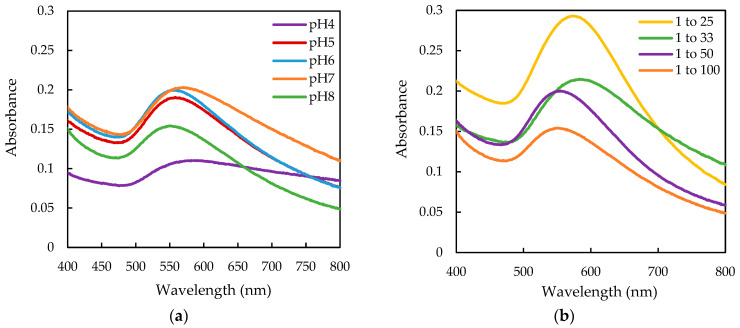
Ultraviolet-visible (UV-vis) spectrum of gold nanoparticles (Au) synthesized with fruit extract (AuKPs) at (**a**) different pH values and (**b**) different ratios of Au^3+^/fruit extract (*w*/*w*) at pH 8.

The impact of pH and the ion-to-extract ratio on the formation of AuNPs synthesized using *T. ferdinandiana* fruit extract (referred to as AuKPs) was different to the leaf extract. The expected colour change from yellow to deep red, indicative of AuNP formation, was not observed for any of the tested pH levels and ratios. Instead, all solutions turned black. The UV-vis spectrum of AuKPs at different pH and different ratios is presented in Figure 3a,b, respectively. The spectra show broader absorption peaks with low intensities, with absorption peaks appearing at longer wavelengths, ranging from 550 nm to 580 nm, which is outside the typical absorption range for AuNPs. DLS analysis revealed that the mean particle size of AuKPs ranged from 140 nm to 200 nm, with a PDI between 0.7 and 1.4. Even though the 1:25 ratio displayed a relatively narrow, higher-intensity peak in the UV-visible spectrum, the DLS results indicated a larger particle size and a PDI greater than 1.0.

**Figure 4:**
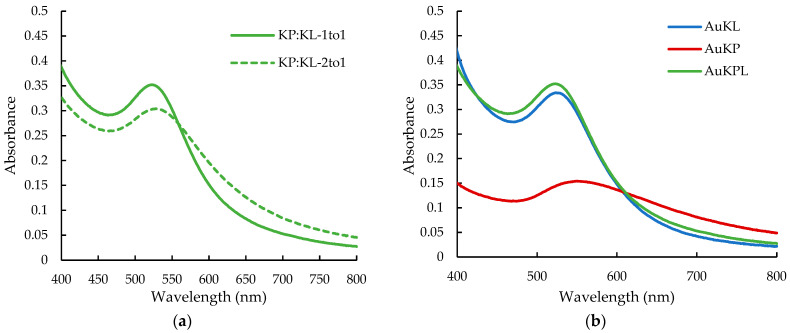
Ultraviolet-visible (UV-vis) spectrum of (**a**) gold nanoparticles synthesized with leaf extract (KL) and fruit extract (KP) mixture (AuKPLs) at different KP/KL extract ratios at pH 8; (**b**) optimized gold nanoparticles prepared at pH 8.

Due to the ineffective synthesis of AuNPs using fruit extracts alone, a combination of leaf extracts (KL) and fruit extracts (KP) was utilized to synthesize a new set of AuNPs, termed AuKPLs (gold nanoparticles synthesized with leaf and fruit extract mixture). As the optimal condition for yielding the smallest AuKLs was pH 8 with ion-to-extract ratios of 1:25, AuKPL synthesis was confined to pH 8 and ion-to-extract ratios of 1:25. With these conditions, two different fruit-to-leaf extract ratios, 1:1 and 2:1, were evaluated to test the influence of mixing leaf and fruit extracts. The UV-Vis spectrum in Figure 4a indicates a KP/KL ratio of 1:1, resulting in a narrower absorption band at 523 nm. AuKPL nanoparticles produced at this ratio have a smaller particle size of 35.3 nm and a lower PDI of 0.368 compared to KP/KL—2:1. The UV spectra of the three optimized AuNPs are compared in Figure 4b.

The zeta potential values in Table 1 for the synthesized AuKLs, AuKPs, and AuKPLs at optimal conditions indicate that all samples exhibited a negative surface charge.

**Figure 5:**
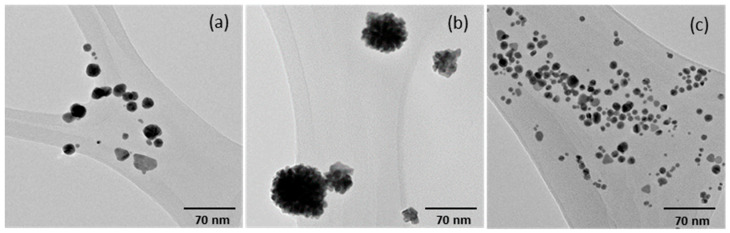
Transmission electron microscopy (TEM) images of (**a**) Gold nanoparticles (AuNPs) synthesized with leaf extract (AuKLs), (**b**) AuNPs synthesized with plum extract (AuKPs), and (**c**) AuNPs synthesized with leaf and fruit extract mixture (AuKPLs), synthesized from *Terminalia ferdinandiana* extracts.

TEM analysis revealed that the optimized AuKL and AuKPL nanoparticles were well distributed in solution, with the majority exhibiting spherical or near-spherical morphologies with few triangular shapes, having average diameters of 14.1 ± 5.8 nm and 8.0 ± 3.8 nm, respectively (Figure 5a,c). Both AuKLs and AuKPLs displayed good dispersion in the reaction medium, with no visible signs of agglomeration. In contrast, the TEM image of AuKPs in Figure 5b showed the formation of agglomerated nanoparticles, with larger sizes averaging 56.1 ± 20.7 nm. The hydrodynamic diameters of the AuNPs obtained from DLS analysis were larger than those measured by TEM. This difference arises because DLS measures the hydrodynamic diameter, which includes the phytochemical coating and the surrounding layer of water molecules, whereas TEM provides an accurate measurement of only the inorganic core of the nanoparticles [38,39].

### 3.2. Biological Activity of AuNPs

As AuNPs did not result in well-formed NPs with fruit extract (AuKPs), this study focused on assessing only the optimized AuKL and AuKPL nanoparticles for their antioxidant and anticancer activities and compared them with the corresponding crude extracts: KL, KP, and KPL (leaf and fruit extract mixture). For comparative analysis, the concentrations of the crude extracts used in all assays were equivalent to the extract levels incorporated into the nanoparticles. This ensured a direct comparison between the bioactivities of the nanoparticles and their respective crude extracts across all tests.

**Figure 6:**
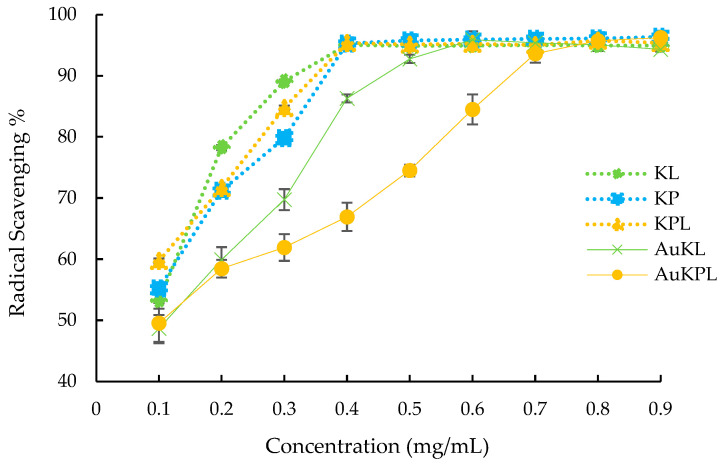
Antioxidant activity of *Terminalia ferdinandiana* extracts and synthesized gold nanoparticles (AuNPs). Results are presented as mean ± SD. AuKL: AuNPs synthesized with leaf extract; AuKPL: Gold nanoparticles synthesized with leaf extract (KL) and fruit extract (KP) mixture (KPL).

The antioxidant capacities of the *T. ferdinandiana* extracts and the synthesized AuNPs are presented in Figure 6. Statistical analysis showed that the *p* values for both the AuNPs and the *T. ferdinandiana* extracts were below 0.05 (*p* < 0.001), confirming that the concentration-dependent increases in DPPH radical-scavenging activity were statistically significant. All extracts and AuKPLs exhibited ≥50% radical-scavenging activity at the lowest concentration tested (0.1 mg/mL). Furthermore, all *T. ferdinandiana* extracts achieved >90% DPPH inhibition at 0.4 mg/mL, whereas the AuKPLs reached ≥90% antioxidant activity from 0.7 mg/mL, while the AuKLs displayed 90% inhibition at concentrations ≥0.5 mg/mL. Collectively, these findings demonstrate the strong in vitro antioxidant potential of the synthesized AuNPs.

**Figure 7:**
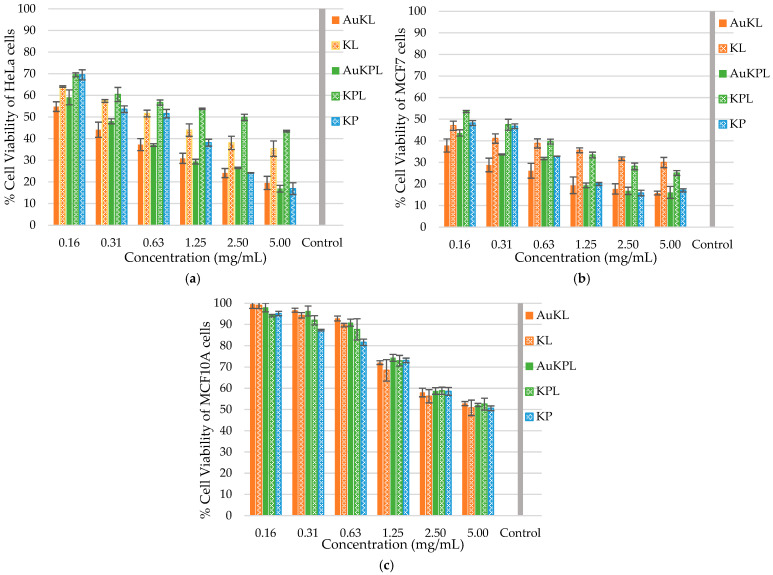
Cytotoxic effect of *Terminalia ferdinandiana* extracts, AuKLs (gold nanoparticles (AuNPs) synthesized with leaf extract (KL)), and AuKPLs (AuNPs synthesized with leaf and fruit extract) on (**a**) HeLa (human cervical carcinoma), (**b**) MCF7 (breast cancer), and (**c**) MCF10A (normal mammalian breast) cells by MTT (3-(4,5-dimethylthiazol-2-yl)-2,5-diphenyltetrazolium bromide) assay. The results were analyzed using two-way ANOVA in GraphPad Prism 10. Results are presented as the mean ± SD of three technical replicates from each of three independent trials.

The biocompatibility of AuNPs is a crucial determinant influencing their potential in biomedical applications, as cytotoxicity towards normal cells can limit their therapeutic efficacy. In order to address this, we evaluated the cytotoxicity of AuKL, AuKPL, KL, KP, and KPL crude extracts on MCF10A cell lines along with cancer cell lines MCF7 and HeLa. The cytotoxicity of various concentrations of *T. ferdinandiana* extracts and AuKL and AuKP nanoparticles against MCF7, HeLa cancer cell lines, and MCF10A normal cells is shown in Figure 7.

Figure 7a,b demonstrate that both *T. ferdinandiana* extracts and the synthesized AuNPs exhibited significant inhibitory effects on the proliferation of MCF7 and HeLa cancer cells. The results showed that the potential growth inhibition of cancer cells increased with the increase in concentration of NPs and extracts. The growth inhibition of MCF7 gradually increased from 62.2% to 84.2% for AuKLs and 56.4% to 83.9% for AuKPLs, with an increase in concentration from 0.16 mg/mL to 5.00 mg/mL. The growth inhibition towards HeLa cells increased from 45.2% to 80.4% for AuKLs and 41.0% to 83.1% for AuKPLs.

Notably, Figure 7a,b showed that the percentage of viable cells treated with extracts was significantly higher (*p* < 0.0001) compared to those treated with AuKLs and AuKPLs, at equivalent concentrations. The largest difference in growth inhibition between AuNPs and extracts towards MCF7 was shown at a concentration of 1.25 mg/mL (AuKLs showed a 16.2% increase, while AuKPLs showed a 14.1% increase), and the largest difference towards HeLa was shown at a concentration of 5.00 mg/mL (nearly 16% with AuKLs and nearly 26% with AuKPLs). These results clearly indicate that both AuKLs and AuKPLs displayed significantly higher cytotoxicity (*p* < 0.0001) against MCF7 cells compared to HeLa cells. AuKLs and AuKPLs showed similar inhibitory effects against MCF7 and HeLa cells across all tested concentrations.

Although 25–45% cytotoxicity was observed at higher concentrations (1.25–5.00 mg/mL), at lower concentrations, no significant toxicity was seen on MCF10A normal cells treated with either *T. ferdinandiana* extracts or the synthesized AuNPs (Figure 7c). The cell viability of MCF10A normal cells was higher than 90% up to a concentration of 0.63 mg/mL of AuKLs and AuKPLs, and gradually decreased to 52% with the increase in concentration until 5.00 mg/mL. These results further confirm the biocompatibility of both the AuKLs and AuKPLs with normal cells.

## 4. Discussion

Numerous studies have highlighted the presence of various phytochemicals, including tannins, flavonoids, alkaloids, ascorbic acid, and other polyphenolic compounds in solvent extracts from *T. ferdinandiana* leaves and fruit [33,40,41,42]. Our prior research identified a total of 126 compounds in *T. ferdinandiana* aqueous extracts using UPLC-MS (both positive and negative ion modes). Notably, ascorbic acid was abundant in the fruit extract, while the leaf extract was rich in a diverse range of tannins, flavonoids, and alkaloids. These findings suggest that a combination of phytochemicals present in *T. ferdinandiana* aqueous extracts may serve as an effective reducing and stabilizing agent in the conversion of Au^3+^ to Au^0^, facilitating the synthesis of AuNPs with enhanced biological properties.

The synthesis process of AuNPs is rapid, as evidenced by the distinct colour change in the solution from pale yellow to ruby red, due to the SPR phenomenon, a hallmark of AuNP formation [43]. Additionally, the UV-Vis spectra of the synthesized *T. ferdinandiana*-AuNPs showed an absorbance peak between 520 nm and 535 nm, further confirming the formation of AuNPs. This result is consistent with previous studies, such as those by Khandanlou et al. [25,26], demonstrating the effectiveness and reliability of plant extracts in synthesizing AuNPs through green methods.

The size and surface chemistry significantly affect the absorption, biodistribution, and pharmacokinetics of nanoparticles as drug carriers, making them critical factors in biomedical applications [44]. The pH plays a pivotal role in regulating the reduction of metal ions, the formation of nucleation centres, and the growth of nanoparticles [16]. Previous studies have shown that nanoparticle size decreases with increasing pH [45], as fewer NPs form at lower pH, resulting in larger particles. The UV-visible spectra, PDI, and hydrodynamic diameter results recorded in our study reveal that the pH of the reaction mixture significantly influences both the size and dispersion of AuNPs. The slight blue shift in the UV-visible spectra with the increase in pH indicates the reduction in particle size [46] of AuKLs. This trend aligns with the DLS results (Figure 2a). The formation of larger NPs at lower pH may be due to uncontrolled nucleation and aggregation [47], as well as possible degradation or inactivation of bioactive molecules at acidic conditions [25]. This work demonstrates that a pH of 8 yields small and stable AuKLs with an average size of 21.1 nm and a PDI of 0.17, signifying a well-dispersed distribution. These results are consistent with previous studies, such as those using olive leaf (*Olea europaea*) extract AuNPs, which also found that larger particles formed at lower pH (3.3) compared to basic conditions (pH 9.6) [48].

The results of this study revealed that different Au ion-to-leaf extract ratios influenced the size and size distribution of AuKLs. The UV-vis spectra in Figure 1b showed that the 1:25 Au^3+^-to-leaf extract ratio resulted in a narrow absorption peak with the highest intensity, indicating well-defined nanoparticles. These spectral observations were corroborated by DLS measurements with small particle size and low PDI at this ratio. The presence of larger NPs above or below the optimum ratio could be due to insufficient reactant in the mixture leading to uncontrolled nucleation, and aggregation of AuNPs [22].

Despite the well-defined synthesis of AuNPs using *T. ferdinandiana* leaf extract, AuNPs with fruit extract (AuKPs) turned black instead of ruby red. In the UV-vis spectra, broad absorption peaks with low intensities appeared around 550 nm, indicating the formation of polydisperse (PDI ranging from 0.7 to 1.4) and larger nanoparticles (ranging from 140 nm to 200 nm) under all tested parameters, suggesting that the fruit extract lacks the necessary phytochemicals for capping and stabilizing the nanoparticles. LC-MS results (Table S1) also demonstrated a higher phytochemical content in the leaf extract compared to the fruit extract. Specifically, the leaf extract is rich with phytochemicals with capping and stabilizing abilities, such as amino acids [49,50,51,52,53,54,55,56], lignin [57], flavonoids [58,59], polyphenols, and tannins [60,61], which facilitate the synthesis of well-homogenized, stable nanoparticles. In contrast, the fruit extract was deficient in these compounds.

In order to address the lack of capping and stabilizing agents in the fruit extract, combining the leaf extract with the fruit extract resulted in nanoparticles (AuKPLs) with a narrower UV-vis absorption band, smaller particle size, and lower PDI than those obtained with fruit extract alone.

Interestingly, we found that a higher proportion of leaf extract is necessary for the successful formation of AuKPLs compared to AgKPLs in our prior work [12]. More phytochemicals in the fruit extract are required for the reduction of Au^3+^ ions to Au^0^, compared to Ag^+^ to Ag^0^ reduction.

AuNPs show a negative zeta potential, which indicates effective stabilization. Phytochemicals present in extracts contribute to the formation of a protective layer around the AuNPs, thereby preventing agglomeration and enhancing their stability [25,26,38]. The surface charge of AuNPs also significantly impacts their toxicity and interactions with biological systems. The presence of a negative surface charge in AuNP suspensions not only signifies a high level of stability but also implies a lower level of toxicity towards normal cells and preferential accumulation in tumour cells, whereas positively charged and neutral AuNPs are absorbed by all cell types at the same rate [44,62].

The total *T. ferdinandiana* leaf and fruit extracts contain various phytochemicals with outstanding antioxidant activities [30,32,63,64,65]. The DPPH assay in this study aimed to determine whether the synthesized AuNPs retained the antioxidant properties of the original *T. ferdinandiana* extracts [32]. Statistical analysis indicated a significant correlation (*p* < 0.0001) between DPPH percentage inhibition and the concentrations of both AuNPs and *T. ferdinandiana* extracts, up to a 90% inhibition threshold. These findings confirm that the synthesized AuNPs effectively retained the antioxidant properties exhibited by the *T. ferdinandiana* extracts.

Prior studies documented the potent anticancer properties of *T. ferdinandiana* extracts using different solvents [41,66]. Ramadhania et al. [67] used *T. ferdinandiana* fruit extract to synthesize ZnO nanoparticles which exhibit significant cytotoxicity against human lung adenocarcinoma (A549) cells.

In our study, both AuKLs and AuKPLs synthesized with the aqueous extract of *T. ferdinandiana* exhibited anticancer efficacy against the tested cancer cell lines, higher than that of the crude extracts. AuKLs and AuKPLs exhibited substantial anticancer efficacy against MCF7 breast cancer cells compared to HeLa cancer cells, suggesting their superiority in the treatment of breast cancer cells. Additionally, 0.63 mg/mL is the optimal concentration where AuNPs show the highest cytotoxicity towards both cancer cells while showing the lowest cytotoxicity towards normal cells.

While the anticancer efficacy of biosynthesized AuNPs is complex and not completely understood, biosynthesized AuNPs are regarded as possible vehicles for phytocomponents that possess anticancer properties [26]. *T. ferdinandiana* AuNPs demonstrated significant cytotoxicity in MCF7 and HeLa cells, likely due to the synergistic effects of biomolecules such as phenols [68], alkaloids [69,70], tannins [71,72,73], and flavonoids [74,75,76] which possess anti-proliferative activities capped on the AuNPs.

AuNPs show shape and size-dependent cytotoxic activity against different cancer cells [77,78,79].

For example, Al-Khedhairy et al. [80] investigated the size-dependent cytotoxicity of AuNPs (size 10–15 nm, 20–10 nm, and 45 nm) on liver cancer cells and found that smaller AuNPs had a more significant cytotoxic effect on HepG2 (human liver cancer) cells. In this study, AuNP synthesis, which shows anticancer properties, also possesses a hydrodynamic size range of 20–30 nm. That may be due to smaller AuNPs exhibiting increased biological and chemical activity and higher surface-area-to-volume ratio, allowing for enhanced macromolecule adsorption and targeted cell interaction through surface functionalization [25]. The cytotoxicity of AuNPs also depends on the dose of nanoparticles [81,82,83]. Khandanlou et al. [25] also reported dose-dependent cytotoxicity of AuNPs synthesized using *Backhousia citriodora* leaf extract against both MCF7 and HepG2 cancer cells. In our study, AuNPs synthesized with *T. ferdinandiana* show dose-dependent cytotoxicity.

## 5. Conclusions

This study represents the first effort to synthesize stable AuNPs from aqueous extracts of *T. ferdinandiana* and evaluate their cytotoxicity against MCF7 and HeLa cancer cells. The size and distribution of AuNPs were optimized by changing the pH and Au ion-to-extract ratio of the reaction mixture. Following the optimization process, we successfully produced small-sized, well-stabilized and homogenized AuNPs (AuKLs and AuKPLs) at a pH of 8 with a 1:25 Au^3+^-to-extract (*w*/*w*) ratio, utilizing *T. ferdinandiana* leaves and a combination of leaf and fruit extracts at ambient temperature. AuKLs and AuKPLs exhibited spherical or nearly spherical morphologies, with average dimensions of 21.1 nm and 28.2 nm, respectively. The PDI measurements of 0.17 and 0.37 for AuKLs and AuKPLs indicated the good to moderate dispersity of the synthesized AuNPs. The zeta potential values (−80.1 mV and −73.3 mV for AuKLs and AuKPLs, respectively) confirmed the stability of the AuNPs. The findings demonstrate that the fruit extract alone was inadequate for the formation of AuNPs. Nonetheless, including leaf extract into the fruit at a 1:1 ratio in the reaction mixture stabilized the AuNPs, producing well-dispersed, small nanoparticles. This indicates that the desired AuNPs were successfully synthesized highlighting the essential role of the phytochemicals present in the leaf extract for effective capping and stabilization. Furthermore, these results confirm that the fruit extracts are deficient in the phytochemicals necessary to function as capping and stabilizing agents for AuNP synthesis.

AuKLs and AuKPLs demonstrated significant DPPH free radical scavenging activity; thus, the synthesized AuNPs preserved the antioxidant capabilities of *T. ferdinandiana* extracts. In vitro anticancer results from this study indicate that the AuKLs and AuKPLs exhibit higher cytotoxicity against MCF7 and HeLa cancer cells, compared to the plant extract. The comparable cytotoxic effects of AuKLs and AuKPLs suggest that the formation of AuNPs in the mixture is primarily driven by the leaf aqueous extract, with minimal contribution from the fruit aqueous extract. This indicates that the phytochemicals in the fruit extract do not interact effectively with the nanoparticles and do not enhance the cytotoxicity of the AuNPs. Furthermore, both AuKLs and AuKPLs exhibit significant cytotoxicity against MCF7 and HeLa cancer cell lines while demonstrating no cytotoxicity effects towards MCF10A, normal breast cancer cells at low concentrations, indicating their potential as anticancer agents in a targeted and safe manner.

As differences were observed in the formation of AuNPs synthesized using *T. ferdinandiana* leaf and fruit extracts, this variation is likely attributable to the distinct phytochemical profiles of the two extracts. As a subsequent line of investigation, we will examine the specific phytochemicals involved and identify which compounds preferentially interact with and adsorb onto the nanoparticle surfaces.

## Figures and Tables

**Table 1:** Zeta-potential, polydispersity index (PDI) value, and hydrodynamic diameter of optimized gold nanoparticles (AuNPs).

AuNPs	Zeta Potential (mV)	PDI	Hydrodynamic Diameter (nm)
AuKL *s*	−80.1	0.17	21.1
AuKPs	−71.8	0.70	144
AuKPLs	−73.3	0.37	28.2

AuKLs: Gold nanoparticles (NPs) synthesized with leaf extract; AuKPs: Gold nanoparticles synthesized with fruit extract; AuKPLs: AuNPs synthesized with leaf and fruit extract mixture; AuNPs: Gold nanoparticles.

## Data Availability

All data generated or analyzed during this study are included in the published article.

## Supplementary Materials

The following supporting information can be downloaded at: https://www.mdpi.com/article/10.3390/life15121829/s1. Table S1. LCMS metabolomic profiling of *T. ferdinandiana* leaf and fruit water extracts in ESI (−) and ESI (+) mode.

## Abbreviations

The following abbreviations are used in this manuscript:

A549	Human lung adenocarcinoma
Ag	Silver
AgKL	Silver nanoparticles synthesized with leaf extract
AgKP	Silver nanoparticles synthesized with fruit extract
AgKPL	Silver nanoparticles synthesized with leaf and fruit extract mixture
ANOVA	Analysis of variance
Au	Gold
Au^3+^	Metallic gold ions
AuKL	Gold nanoparticles synthesized with leaf extract
AuKP	Gold nanoparticles synthesized with fruit extract
AuKPL	Gold nanoparticles synthesized with leaf and fruit extract mixture
AuNP	Gold nanoparticles
CDU	Charles Darwin University
CO_2_	Carbon dioxide
DLS	Dynamic light scattering
DMSO	Dimethyl sulfoxide
DPPH	2,2 diphenyl-1-picrylhydrazyl
FBS	Fetal bovine serum
GA-1000	Gentamicin-amphotericin
HAuCl4	Chloroauric acid
HeLa	Human cervical carcinoma
HepG2	Human liver cancer cell line
HPW	High-purity water
KL	Leaf extract
KP	Fruit extract
KPL	Leaf and fruit extract mixture
LC-MS	Liquid chromatography-mass spectroscopy
MCF7	Breast cancer cell line
MCF10A	Normal mammalian breast cell line
MEBM	Mammary epithelial cell growth basal medium
MEM	Minimum Eagle’s medium
MTT	3-(4,5-dimethylthiazol-2-yl)-2,5-diphenyltetrazolium bromide
NP	Nanoparticles
NSW	New South Wales
NT	Northern Territory
OD	Optical density
PBS	Phosphate-buffered saline
PDI	Polydispersity index
Qld	Queensland
QUT	Queensland University of Technology
RPMI	Roswell Park Memorial Institute
SD	Standard deviation
SPR	Surface plasmon resonance
TEM	Transmission electron microscopy
UPLC-MS	Ultra-performance liquid chromatography-mass spectrometry
USA	United States of America
UV	Ultraviolet
UV-vis	Visible ultraviolet
