# Supplementary material for: Biofabrication of Terminalia ferdinandiana-Conjugated Gold Nanoparticles and Their Anticancer Properties
Source: Life (Basel). 2025 Nov 28;15(12):1829. doi: 10.3390/life15121829 (PMC12734994; doi:10.3390/life15121829)
Supplement: Supplementary file 1 [file life-15-01829-s001.zip › life-4002634-supplementary.pdf]

Biofabrication of *Terminalia ferdinandiana*-Conjugated Gold Nanoparticles and their Anticancer Properties

Weerakkodige Hansi Sachintha Alwis[1], Vinuthaa Murthy[1*], Hao Wang[1], Roshanak Khandanlou[1], Richard Weir[2]

[1] Faculty of Science and Technology, Charles Darwin University, Australia

[2] Berrimah Veterinary Laboratory, Department of Agriculture and Fisheries, Darwin, NT, Australia,

*Corresponding author:

Dr. Vinuthaa Murthy, Faculty of Science and Technology, Charles Darwin University, Casuarina, Northern Territory, Australia, 0810

Phone number: +61 889466794, Email: vinuthaa.murthy@cdu.edu.au

Supplementary information

Table S1. LCMS metabolomic profiling of *T. ferdinandiana* leaf and fruit water extracts in ESI (−) and ESI (+) mode

RT min	m/z	Adduct	Formula	Tentative ID	Class	Log2FC (F vs. L)
**0.714**	195.051	[M-H]-	C6H12O7	D-Gluconic acid	Sugar acids and derivatives	1.2
**0.722**	165.040	[M-H]-	C5H10O6	Arabinonic acid	Sugar acids and derivatives	0.3
**0.734**	215.033	[M+Cl]-	C6H12O6	Inositol	Sugar alcohol and derivatives	-0.6
**0.751**	104.108	[M]+	C5H13NO	Choline	Cholines	-0.5
**0.786**	116.071	[M+H]+	C5H9NO2	Proline	Amino acids	-1.5
**0.792**	177.040	[M-H]-	C6H10O6	L-Gluconolactone	Gamma butyrolactones	2.1
**0.798**	387.112	[M+CHO2]-	C12H22O11	Sucrose	Disaccharides	2.8
**0.818**	133.015	[M-H]-	C4H6O5	Malic acid	Beta hydroxy acids and derivatives	-0.7
**0.826**	360.150	[M+NH4]+	C12H22O11	Trehalose	Disaccharides	2.1
**0.851**	300.107	[M+K]+	C9H19N5O4	Arginyl-Serine	Dipeptides	-2.4
**0.907**	175.026	[M-H]-	C6H8O6	Vitamin C; ascorbic acid	Butenolides	4.3
**0.962**	177.039	[M+H]+	C6H8O6	Vitamin C; ascorbic acid*	Butenolides	2.8
**1.003**	191.020	[M-H]-	C6H8O7	Citric acid	Tricarboxylic acids and derivatives	-0.6
**1.014**	104.107	[M]+	C5H13NO	Choline*	Cholines	-1
**1.047**	130.087	[M+H]+	C6H11NO2	DL-Pipecolinic acid	Alpha amino acids	-1.2
**1.076**	133.014	[M-H]-	C4H6O5	Malic acid*	Beta hydroxy acids and derivatives	-0.4
**1.108**	234.133	[M+H]+	C10H19NO5	Mevalonic-GABA	Amino acids and derivatives	2.3
**1.161**	146.092	[M+H]+	C5H11N3O2	4-Guanidinobutanoic acid	Gamma amino acids and derivatives	-0.2
**1.213**	129.019	[M-H]-	C5H6O4	Itaconic acid	Fatty acyls	-1
**1.22**	205.035	[M-H]-	C7H10O7	2-Methylcitric acid	Tricarboxylic acids and derivatives	-0.8
**1.224**	229.154	[M+H]+	C11H20N2O3	2-amino-4-methylpentanoyl pyrrolidine-2-carboxylate	Amino acids and derivatives	-0.6
**1.267**	147.029	[M-H]-	C5H8O5	D-(-)-Citramalic acid	Fatty acyls	1.1
**1.296**	500.102	[2M+ACN+H]+	C5H12NO7P	5-Phosphoribosylamine	Pentose phosphates	1.1

**1.299**	351.056	[M-H]-	C12H16O12	a-L-threo-4-Hex-4-enopyranuronosyl-D-galacturonic acid	Sugar acids and derivatives	4.5
**1.396**	117.019	[M-H]-	C4H6O4	Succinic acid	Dicarboxylic acids and derivatives	-1.4
**1.503**	615.059	[2M+K]+	C12H16O4S2	Malotilate	Dicarboxylic acids and derivatives	3.8
**1.504**	599.085	[2M+Na]+	C12H16O4S2	Malotilate*	Dicarboxylic acids and derivatives	3.8
**1.508**	404.118	[M+ACN+Na]+	C12H20O11	3'-Ketolactose	Disaccharides	2.2
**1.547**	132.102	[M+H]+	C6H13NO2	Isoleucine	Alpha amino acids	-2.3
**1.623**	104.107	[M]+	C5H13NO	Choline*	Cholines	-0.2
**1.677**	116.070	[M+H]+	C5H9NO2	Proline*	Amino acids	-0.3
**1.691**	175.025	[M-H]-	C6H8O6	D-(-)-Isoascorbic acid	Butenolides	3.9
**1.719**	351.056	[M-H]-	C6H8O6	D-(-)-Isoascorbic acid*	Butenolides	4.1
**1.77**	234.133	[M+ACN+H]+	C8H16O5	3,4-di-O-methyl-rhamnose	Hexoses	2.3
**1.792**	229.154	[M+H]+	C11H20N2O3	Isoleucylproline	Dipeptides	-0.3
**1.792**	191.019	[M-H]-	C6H8O7	Citric acid	Organic acids	1.1
**1.795**	439.171	[M+Na]+	C23H28O7	Virgatusin	7,7' epoxylignans	-1
**1.857**	332.134	[M+H]+	C14H21NO8	5'-O-beta-D-Glucosylpyridoxine	Carbohydrate and conjugates	-2.2
**1.875**	123.055	[M+H]+	C6H6N2O	Niacinamide	Pyridines and derivatives	-0.6
**1.916**	228.086	[M+H]+	C10H13NO5	L-Arogenic acid	L-alpha-amino acids	-1.5
**1.937**	243.050	[M-H]-	C10H12O7	1-O-Galloylglycerol	Galloyl esters	3.5
**1.96**	331.067	[M-H]-	C13H16O10	beta-Glucogallin	Tannins	-2
**1.977**	439.171	[M+Na]+	C23H28O7	Epimagnolin	Furanoid lignans	-1
**2.002**	296.133	[M+H]+	C12H17N5O4	N-6-(2-Hydroxyethyl)-Adenosine	Purine nucleosides	-0.7
**2.05**	229.154	[M+H]+	C11H20N2O3	Leucylproline	Dipeptides	-0.4
**2.06**	182.045	[M-H]-	C8H9NO4	4-Pyridoxic acid	Pyridines and derivatives	3
**2.269**	318.081	[M+Na]+	C10H17NO9	(S)-malyl alpha-D-glucosaminide	Alpha amino acids and derivatives	3.3
**2.301**	213.004	[M-H]-	C8H6O7	3,4,6-trihydroxybenzene-1,2-dicarboxylic acid	Hydrozybenzoic acid derivatives	-2.2
**2.423**	314.091	[M+H]+	C11H15N5O4S	Methylthioadenosine Sulfoxide	Nucleoside and nucleotide analogs	-4

**2.443**	281.031	[M-H]-	C12H10O8	2-O-Caffeoyltartronic acid	Coumaric acids and derivatives	-2.6
**2.456**	331.066	[M-H]-	C13H16O10	beta-Glucogallin	Galloyl esters	2.1
**2.5**	225.003	[M-H]-	C10H10O2S2	3,3'-Dithiobis[2-methylfuran]	Heteroaromatic compounds	-0.5
**2.515**	166.087	[M+H]+	C9H11NO2	Phenylalanine	Amino acids and derivatives	-3.1
**2.578**	527.160	[M-H]-	C24H32O11S	17-beta-estradiol 3-sulfate-17-(beta-D-glucuronide)	Steroidal glycosides	4.2
**2.652**	131.035	[M-H]-	C5H8O4	Ethylmalonic acid	Fatty acyls	2.7
**2.68**	301.056	[M-H]-	C12H14O9	5-Galloyloxy-3,4-dihydroxypentanoic acid	Galloyl esters	3.2
**2.723**	328.139	[M+H]+	C15H21NO7	Sesbanimide A	Piperidinediones	-1.2
**2.735**	175.024	[M-H]-	C6H8O6	D-(-)-Isoascorbic acid*	Butenolides	3.3
**2.85**	281.030	[M-H]-	C12H10O8	2,5,6,8-tetrahydroxy-3,7-dimethoxy-1,4-dihydronaphthalene-1,4-dione	Phenolic acids	-3.6
**2.897**	289.091	[M+H]+	C12H16O8	Glucosylisomaltol	O-glycosyl compounds	0.1
**2.971**	291.014	[M-H]-	C13H8O8	Phyllanthusiin E	7,8-dihydroxycoumarins	-4.1
**2.991**	323.004	[M+K-2H]-	C7H15N2O8P	Glycineamideribotide	Glycinamide ribonucleotides	-3.3
**2.999**	301.056	[M-H]-	C12H14O9	Pyrogallol-2-O-glucuronide	Phenolic glycosides	-0.8
**3.039**	220.118	[M+H]+	C9H17NO5	Pantothenic acid	Secondary alcohols	0.3
**3.058**	545.114	[M-H]-	C29H22O11	[2-[3-(3,4,5-trihydroxybenzoyl)oxyphenyl]-3,4-dihydro-2H-chromen-3-yl] 3,4,5-trihydroxybenzoate	Gallic acid and derivatives	-1.5
**3.073**	611.124	[M-H]-	C26H28O17	Myricetin 3-Sambubioside	Flavonoid glycoside	-1.6
**3.149**	206.139	[M+H]+	C9H19NO4	Pantothenol	N-acyl amines	-2.4
**3.153**	783.067	[M-H]-	C34H24O22	5'-Desgalloylstachyurin	Hydrolyzable tannins	-2.7
**3.192**	180.101	[M+H]+	C10H13NO2	Fusaric acid	Amino fatty acids	1.2
**3.306**	165.056	[M-H]-	C9H10O3	Phloretic Acid	Phenylpropanoic acid	0.1
**3.459**	285.061	[M-H]-	C12H14O8	Uralenneoside	p-Hydroxybenzoic acid alkyl esters	-2
**3.501**	205.097	[M+H]+	C11H12N2O2	Tryptophan	Indolyl carboxylic acids and derivatives	-1
**3.546**	307.01	[M-H]-	C13H8O9	3-(5-Acetyl-2-carboxyfuran-3-yl)furan-2,5-dicarboxylic acid	Coumarin and derivatives	-3.3
**3.634**	122.096	[M+H]+	C8H11N	Phenylethylamine	Phenethylamines	-3.7
**3.942**	483.078	[M-H]-	C20H20O14	1,6-Digalloyl-beta-D-glucopyranose	Tannins	1
**3.983**	451.051	[M-H]-	C19H16O13	3,4-Hexahydroxydiphenoylarabinose	Hydrolyzable tannins	-4.2

**4.18**	383.131	[M+Na]+	C16H24O9	Deoxyloganic acid	Iridoid O-glycosides	0.9
**4.266**	483.079	[M-H]-	C20H20O14	1,6-Digalloyl-beta-D-glucopyranose*	Tannins	1.7
**4.38**	293.124	[M-H]-	C12H22O8	Ethyl (S)-3-hydroxybutyrate glucoside	Fatty acyl glycosides of mono- and disaccharides	-1.2
**4.498**	483.07	[M-H]-	C20H20O14	1,6-Digalloyl-beta-D-glucopyranose*	Tannins	-1.4
**4.523**	433.040	[M-H]-	C19H14O12	Ellagic acid arabinoside	Hydrolyzable tannins	-3.9
**4.564**	183.029	[M-H]-	C8H8O5	3,4-dihydroxy-5-methoxybenzoic acid	Hydroxy benzoic acid and derivatives	3.1
**4.733**	433.041	[M-H]-	C19H14O12	Ellagic acid arabinoside	Hydrolyzable tannins	-4
**4.854**	169.014	[M-H]-	C7H6O5	Gallic acid	Gallic acids and dervatives	3.1
**4.924**	401.181	[M+H]+	C19H28O9	Corchoionoside B	Fatty acyl glycosides of mono- and disaccharides	-1.6
**5.044**	305.071	[M-H]-	C12H18O7S	(3R,7S)-12-Hso4-Ja	Jasmonic acids	-0.7
**5.045**	95.0127	[M+H-H2O]+	C5H4O3	2-Furoic acid	Furoic acids	4.3
**5.245**	785.083	[M-H]-	C34H26O22	Heterophylliin A	Hydrolyzable tannins	-0.6
**5.317**	406.207	[M+NH4]+	C18H28O9	7-Epi-12-hydroxyjasmonic acid glucoside	Fatty acyl glycosides of mono- and disaccharides	-1.5
**5.354**	273.007	[M-H]-	C10H10O7S	Ferulic acid 4-O-sulfate	Coumaric acid and derviatives	-4.1
**5.43**	633.074	[M-H]-	C27H22O18	Corilagin	Hydrolyzable tannins	0.1
**5.504**	969.083	[M-H]-	C41H30O28	Phyllanthusiin B	Hydrolyzable tannins	-3.6
**5.611**	371.098	[M-H]-	C16H20O10	Dihydroferulic acid 4-O-glucuronide	Phenolic glycosides	-0.2
**5.849**	6350906	[M-H]-	C27H24O18	1,3,6-Trigalloylglucose	Tannins	-2.3
**5.92**	477.103	[M-H]-	C22H22O12	coumaroyl(-6)L-Glc(b)-O-galloyl	Tannins	-0.7
**6.024**	969.083	[M-H]-	C41H30O28	Valolaginic acid	Hydrolyzable tannins	-2.6
**6.205**	925.093	[M-H]-	C40H30O26	Pelargoniin A	Hydrolyzable tannins	-3.7
**6.451**	951.074	[M-H]-	C41H28O27	Sanguiin H11	Hydrolyzable tannins	-0.1
**6.508**	449.109	[M+H]+	C21H20O11	Isoorientin	Flavonoid glycosides	-0.6
**6.568**	953.089	[M-H]-	C41H30O27	Chebulagic acid	Hydrolyzable tannins	-2.9
**6.623**	483.069	[M+Cl]-	C21H20O11	Orientin	Flavonoid glycosides	1.4
**6.644**	937.093	[M-H]-	C41H30O26	Nupharin A	Hydrolyzable tannins	-3.7
**6.697**	493.098	[M-H]-	C22H22O13	Pleurostimin 7-Glucoside	Flavonoid glycosides	-3.8
**6.854**	787.099	[M-H]-	C34H28O22	1,2,4,6-Tetragalloyl-beta-D-glucopyranose	Tannins	-4.5

**7.301**	431.098	[M-H]-	C21H20O10	Isovitexin	Flavonoid glycosides	-1.6
**7.358**	545.198	[M+Na]+	C26H34O11	lariciresinol-glucoside	Lignan glycosides	1.3
**7.394**	599.104	[M-H]-	C28H24O15	Orientin 2''-O-Gallate	Flavonoid glycosides	-1.2
**7.464**	955.105	[M-H]-	C41H32O27	Chebulinic acid	Hydrolyzable tannins	-0.5
**7.719**	197.117	[M+H]+	C11H16O3	Loliolide	Benzofurans	-3.6
**7.937**	520.275	[M+NH4]+	C24H38O11	Eriojaposide A	Fatty acyl glycosides of mono- and disaccharides	-2.1
**8.152**	540.244	[M+NH4]+	C26H34O11	Urolignoside	Lignan glycosides	-2.2
**8.594**	395.204	[M+Na]+	C23H22O6	rotenone	Rotenones	0.9
**8.787**	461.071	[M-H]-	C21H18O12	3-O-Methylducheside A	Hydrolyzable tannins	-0.3
**8.832**	315.014	[M-H]-	C15H8O8	3-O-Methylellagic acid	Hydrolyzable tannins	-4.3
**9.553**	524.140	[M+NH4]+	C23H22O13	Quercetin 3-O-(6''-acetyl-glucoside)	Flavonoid-3-O-glycosides	-2
**9.612**	659.194	[M+FA-H]-	C38H30O8	Spiro-Oxanthromicin A	Phenanthrenes and derivatives	-2.1
**9.795**	557.199	[M+Na]+	C27H34O11	Phillyrin	Terpene lactone	-1.1
**9.881**	689.387	[M+Na]+	C36H58O11	Sericoside	Triterpenoids	1.2
**10.23**	301.035	[M-H]-	C15H10O7	Quercetin	Flavonoid aglycone	2
**10.25**	471.128	[M-H]-	C24H24O10	1,6-Bis-O-(4-Hydroxycinnamoyl)Glucose	Hydroxycinnamic acid and derivatives	-3.5
**11.08**	657.108	[M-H]-	C30H26O17	Eujambin	Flavonoid-3-O-glycosides	-2.8
**12.41**	673.392	[M+Na]+	C36H58O10	Lucyoside N	Triterpene saponins	0.6
**15.37**	532.384	[M+H]+	C28H53NO8	Thermolide E	Diterpene lactones	1.3
